# Long-term progression-free survival in a patient with advanced non-small-cell lung cancer treated with low-dose gefitinib and traditional herbal medicine: A case report

**DOI:** 10.1097/MD.0000000000024292

**Published:** 2021-02-05

**Authors:** Beom-Joon Lee, Kwan-Il Kim, Cheong-Woon Choi, Jong Yeol Kim, Jun-Hwan Lee

**Affiliations:** a Division of Allergy, Immune and Respiratory System, Department of Internal Medicine, College of Korean Medicine, Kyung Hee University, 26 Kyungheedae-ro, Dongdaemun-gu, Seoul, Republic of Korea; b Nowonkyunghee korean medical clinic, 1363, Dongil-ro, Nowon-gu, Seoul, Korea; c Korea Institute of Oriental Medicine; d Korean Medicine Life Science, University of Science & Technology (UST), Campus of Korea Institute of Oriental Medicine, Daejeon, Republic of Korea.

**Keywords:** gefitinib, modified Bojungikki-tang, non-small-cell lung cancer, traditional herbal medicine

## Abstract

**Rationale:**

Gefitinib is a first-line palliative chemotherapy drug used to treat advanced non-small-cell lung cancer (NSCLC) in patients who have an epidermal growth factor receptor (EGFR) mutation. However, approximately two-thirds of NSCLC patients with EGFR-tyrosine kinase inhibitor experience dermatological toxicity. Cutaneous toxicity is usually not life threatening but can necessitate modification or discontinuation of medication in severe cases. In this case, despite a reduction in the dose of gefitinib due to side effects, combined treatment with modified Bojungikki-tang (BJKIT) increased progression-free survival (PFS) in an advanced NSCLC patient.

**Patient concerns:**

An 83-year-old Asian woman presented with chief complaints of chronic cough, dyspnea, weight loss, and anorexia.

**Diagnoses:**

The patient was diagnosed with stage IV NSCLC (T2aN3M1), adenocarcinoma with metastasis to the lymph node, brain, and bone based on image scan and biopsy. An EGFR deletion was detected in exon 19.

**Interventions:**

The patient was treated with gefitinib (250 mg/d) and traditional herbal medicine, modified Bojungikki-tang (BJIKT). However, after 1 year of combination therapy, gefitinib was tapered down to once per week while modified BJIKT was maintained.

**Outcomes:**

A partial response was achieved, but after 3 months severe papulopustular skin rashes developed and became aggravated with time. Thus, the gefitinib dose was reduced. However, the PFS has been maintained for approximately 78 months.

**Lessons:**

Despite the reduction in gefitinib dose due to side effects, the combined treatment of gefitinib and the modified BJIKT has maintained a PFS of over 78 months, indicating that modified BJIKT enhanced the anti-cancer effect of gefitinib in a patient with advanced NSCLC harboring the EFGR mutation, and may have delayed acquired resistance, the main limitation on the efficacy of gefitinib. Further investigations including clinical trials are needed to confirm these effects.

## 1 Introduction

Lung cancer is the most common cancer in both incidence (11.6%) and mortality (18.4%) worldwide.^[1]^ In Korea, the crude incidence rate of lung cancer was 43.9 per 100,000 in 2012, and the non-smoker lung cancer incidence increased from 19.1% in 2004 to 2008 to 25.4% in 2009 to 2012.^[2]^ As early-stage lung cancer is asymptomatic, most patients are diagnosed at an advanced stage and have a poor prognosis.^[3]^ In particular, the prognosis for advanced non-small-cell lung cancer (NSCLC) is very poor, with a median survival of 9.5 months in 2010, an increase of only 1.5 months compared to 2000.^[4]^ The therapeutic strategy for stage IV advanced lung cancer is palliative treatment. The goals of management are to prolong survival and maintain quality of life for as long as possible, while minimizing the side effects due to treatment. If the epidermal growth factor receptor (EGFR) mutation is detected in advanced NSCLC, EGFR-tyrosine kinase inhibitors (TKIs), such as gefitinib, have become standard practice as first-line palliative chemotherapy.^[5]^

Gefitinib is a first-generation EGFR-TKI that is used for patients with NSCLC who have an EGFR exon 19 deletion or exon 21 (L858R) substitution mutation. Gefitinib improves response rates, increases the time to progression and overall survival, and, in particular, shows better effectiveness for Asians, adenocarcinomas, and non-smokers or light smokers.^[6]^ However, it is limited for controlling tumor progression because acquired resistance usually occurs within 6 to 12 months.^[7,8]^ Many attempts have been made to overcome resistance, including using another target agent to bypass the signaling pathway blockade, combination chemotherapy or immunotherapy, or next-generation EGFR-TKI. However, the efficacy of such strategies has been questionable or limited among different clinical trials.^[9]^

In East Asia, including Korea, China, and Japan, traditional herbal medicine (THM) has been commonly used to treat cancer. THM is used in combination with conventional treatments to enhance efficacy and reduce side effects.^[10]^ It has been reported that THM is effective as an adjuvant therapy in patients with NSCLC.^[11]^ In particular, THM combined with EGFR-TKI increases progression-free survival (PFS) and overall survival (OS), improves quality of life, decreases side effects, and delays acquired resistance in patients with advanced NSCLC.^[11–14]^

Bojungikki-tang (BJIKT) (Bu-Zhong-Yi-Qi-Tang in Chinese or Hochu-ekki-to in Japanese) is a traditional herbal prescription widely used in East Asia. In THM, BJIKT has been used to treat complaints of “Qi deficiency” symptoms related to chronic diseases, such as general fatigue, loss of appetite, dyspepsia, spontaneous sweating, restlessness, and slurred speech.^[15]^ It has also been used to treat various lung diseases, including chronic bronchitis, upper respiratory infection, and chronic obstructive pulmonary disease by ameliorating systemic inflammation and improving immunological capacity.^[16–18]^ In addition, BJIKT has been used to treat cancer-related symptoms, such as fatigue, anorexia, and cachexia^[19,20]^ and has anticancer effects itself in several cancers.^[21]^ In particular, experimental and clinical studies suggest that BJIKT is effective as an adjuvant treatment, as it enhances conventional treatments and increases immune function in patients with NSCLC.^[13,22–24]^ However, the effect of BJIKT on survival outcome in advanced NSCLC has not been investigated.

In this case report, we present an elderly patient with stage IV NSCLC who showed a sustained partial response (PR) for over 6 years following a combined treatment of modified BJIKT and a reduced dose of gefitinib.

## 2 Case report

An 83-year-old Asian woman presented to a local clinic in January 2014 with a several-month history of chronic cough with a small amount of white sputum. Cytokeratin-19 and neuron-specific enolase were positive, and she was transferred to a university hospital.

Physical examination revealed dyspnea during exercise and daily activity, anorexia with weight loss to 39 kg, which was 7 kg less than usual, and dizziness, with Eastern Cooperative Oncology Group performance status grade 1. Auscultation revealed vesicular bronchial sounds without crackling or wheezing. She had untreated osteoporosis and had never been a smoker. Her family history was negative.

**Figure 1:**
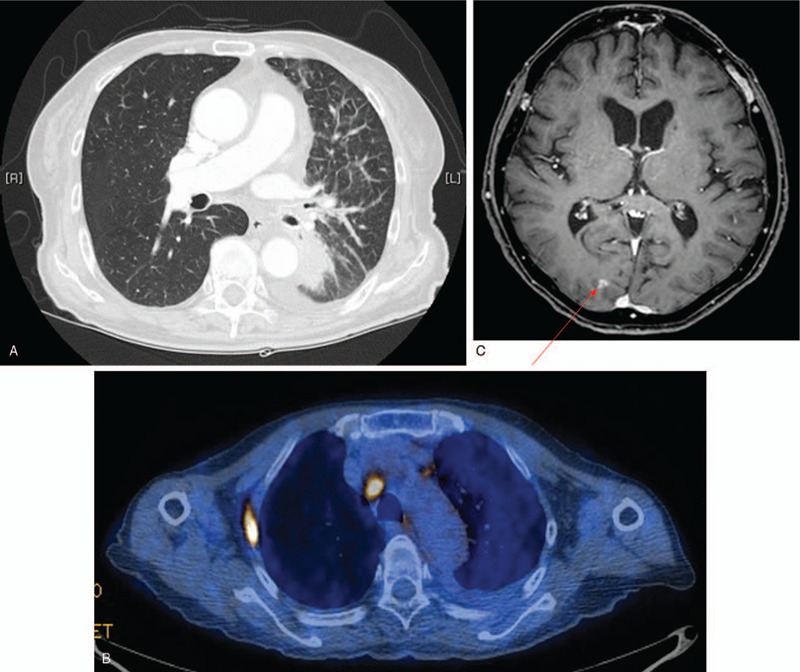
(A) A chest computed tomography scan on January 2, 2014, revealed a mass with a long axis of 3.9 cm in the left lower lobe with lymphangitic metastasis and pleural effusion. (B) A positron emission tomography/CT scan on January 4, 2014, showed bone metastasis in the right third and tenth ribs. (C) A brain magnetic resonance image on January 2, 2014, showing the cortical metastasis. The red arrow indicates a metastatic lesion with high signal intensity.

Chest computed tomography (CT) scan revealed lung cancer in the left lower lobe with lymphangitic metastasis and pleural effusion (Fig. 1A). The longest axis of the mass was 3.9 cm. A subsequent biopsy indicated an NSCLC adenocarcinoma, and a positron emission tomography-CT scan indicated bone metastasis in the right third and tenth ribs (Fig. 1B). Subsequent contrast-enhanced brain magnetic resonance imaging (MRI) revealed cortical metastases (Fig. 1C).

An EGFR deletion was detected in exon 19, but no KRAS mutation was detected. She was finally diagnosed with stage IV NSCLC (T2aN3M1) and brain and bone metastases. Therefore, in February 2014 she was started on 1 tablet of gefitinib (250 mg) per day as palliative chemotherapy. Simultaneously, modified BJIKT (Table 1) was administered 3 times per day, 30 min after every meal based on her symptoms, including dyspnea and general weakness with anorexia.

**Table 1:** Composition of modified Bojungikki-tang (BJIKT).

Herbal name	Scientific name	Part used	Dose (g/d)
Ginseng radix	*Panax ginseng* C.A. Meyer	Root	24
Astragali radix	*Astragalus membranaceus* (Fisch.) Bunge	Root	24
Liriopes radix	*Liriope platyphylla* F.T Wang & Tang	Root	24
Magnoliae obovatae cortex	*Magnolia obovata* Thunb	Bark	16
Zingiberis rhizoma recens	*Zingiber officinale* Roscoe	Rhizome	12
Atractylodis japonicae rhizoma	*Atractylodes japonica* Koidz. Ex Kitam.	Rhizome	8
Angelicae gigantis radix	*Angelica gigas* Nakai	Root	8
Citri reticulati exocarpium et mesocarpium	*Citrus reticulata* Blanco	Ripe pericarp	8
Glycyrrhizae uralensis radix et rhizoma	*Glycyrrhiza uralensis* Fisch.	Root and Rhizome	8
Pinelliae tuber	*Pinellia ternata* (Thunb.) Makino	Tuber	8
Zizyphi fructus	*Zizyphus jujuba var. inermis* Rehder	Fruit	8
Cervi Pantotrichum Cornu	*Cervus nippon* Temminck	Cornu	5
Agastachis Herba	*Agastache rugosa* (Fisch. et Meyer) O. Kuntze	Aerial part	4
Perilla Folium	*Perilla frutescens var. acuta* Kudo	Leaf and twig	4

**Figure 2:**
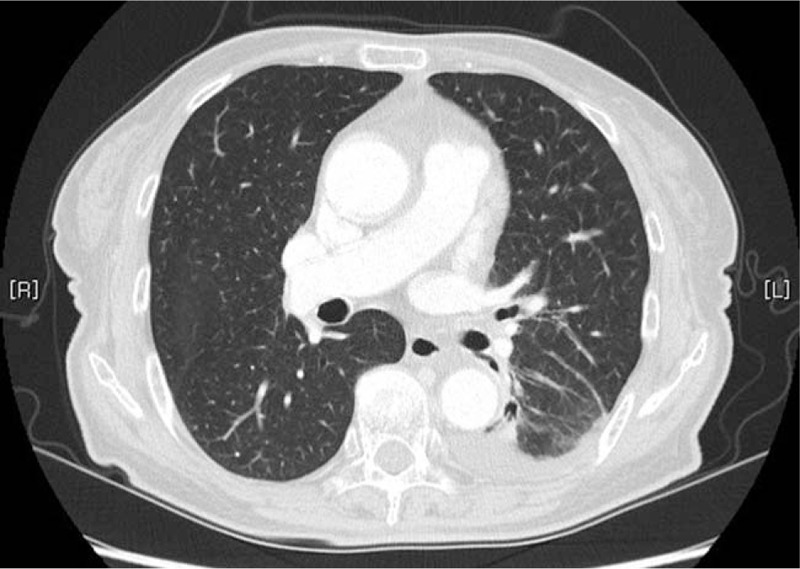
A follow-up chest computed tomography scan on April 17, 2014, indicates a decrease in the mass, with the longest axis, of 1.5 cm, in the left lower lobe and a decrease in the extent of lymphangitic metastasis and pleural effusion.

**Figure 3:**
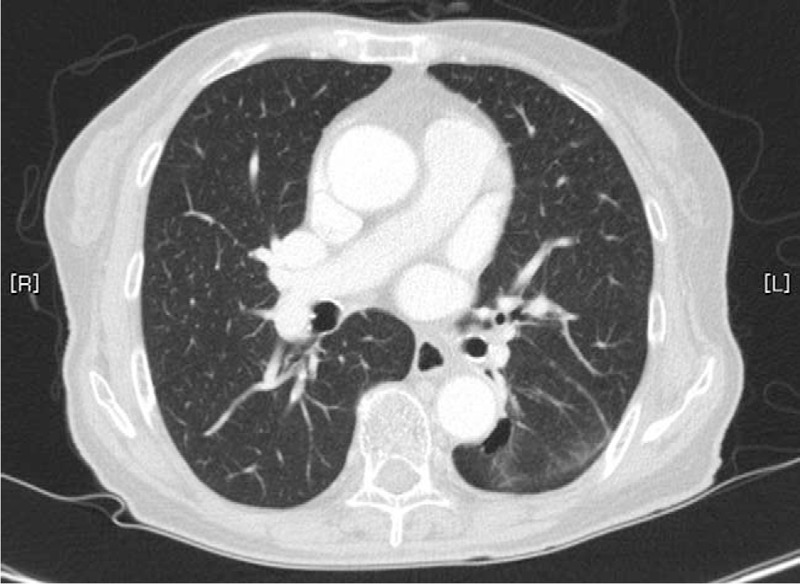
A follow-up chest computed tomography scan obtained on September 17, 2014, revealed a further decrease in the extent of the lymphangitic metastasis and pleural effusion. Thin-wall cavitation with a ground glass opacity is shown.

PR was achieved in April 2014. The chest CT scan revealed an interval decrease in the size of the primary lung cancer in the left lower lobe and a decreased extent of lymphangitic metastasis. The tumor longest axis of the mass had decreased to 1.5 cm, and the diffuse interlobular septal thickening had decreased in the left lung (Fig. 2). In September 2014, a further decrease in lymphangitic metastasis and pleural effusion was observed, and a thin-walled cavity with ground glass opacity started to show (Fig. 3). Her symptoms began to improve gradually, but a mild skin rash and paronychia appeared.

**Figure 4:**
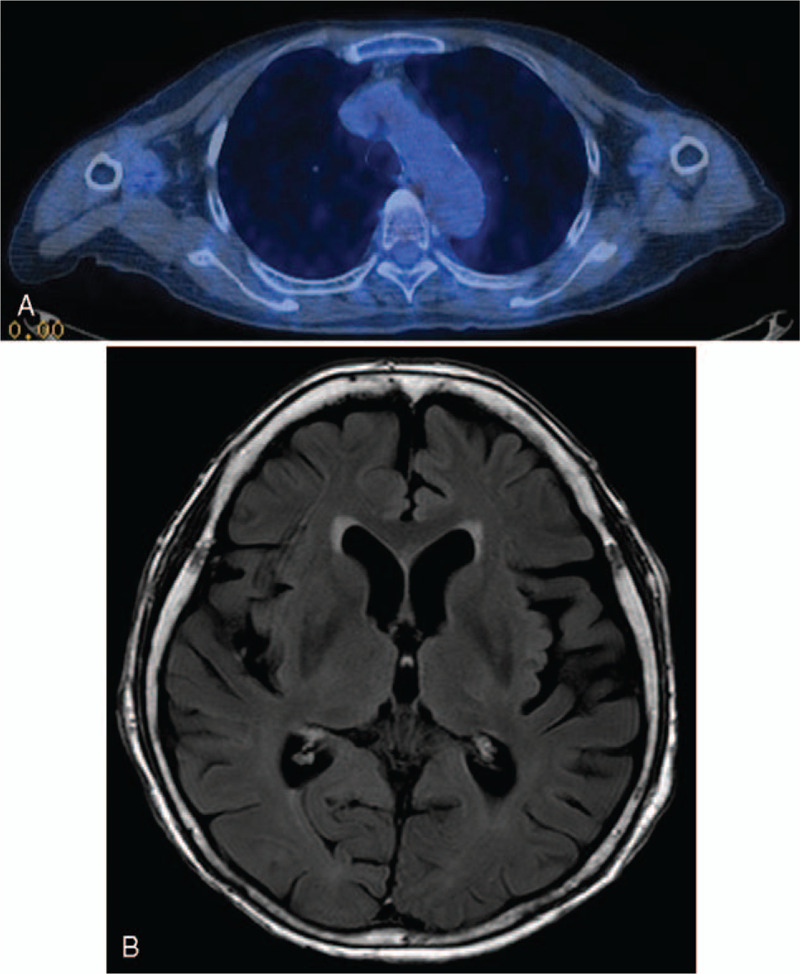
One year after treatment, a follow-up positron emission tomography-computed tomography scan (A) and brain magnetic resonance image (B) on March 18, 2015. The metastatic bone and brain lesions had disappeared.

**Figure 5:**
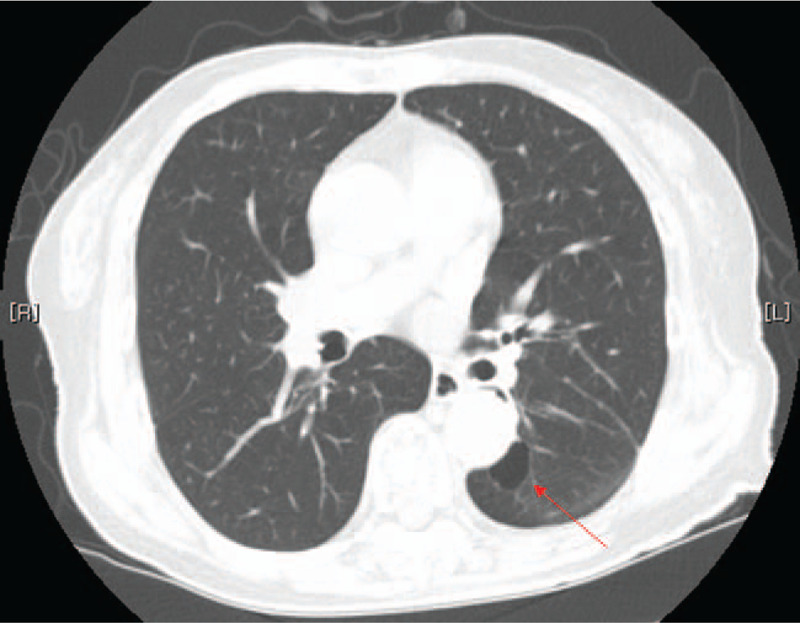
A follow-up chest computed tomography scan on July 31, 2015 revealed that the primary tumor had changed to a thin-walled cavitation.

The PR continued in December 2014. Her symptoms, general weakness, anorexia, and dyspnea were much improved, and her weight decreased to 42.3 kg. However, side effects such as a grade 1 papulopustular skin rash occurred; papules and pustules appeared on her face, neck, and scalp. In March 2015, the PR continued, and a brain MRI showed that the enhanced metastatic lesions had disappeared (Fig. 4AB). However, the skin lesions had worsened to grade 2, paronychia and erosion of the perineal region were aggravated, and irregular scalp thickening with an enlarged lymph node was observed on a brain MRI. Thus, the gefitinib dose was reduced to 2 tablets every 3 days, and the THM was administered at the same dose. In July 2015, a chest CT scan revealed that the primary tumor had changed to a thin-walled cavitation (Fig. 5).

**Figure 6:**
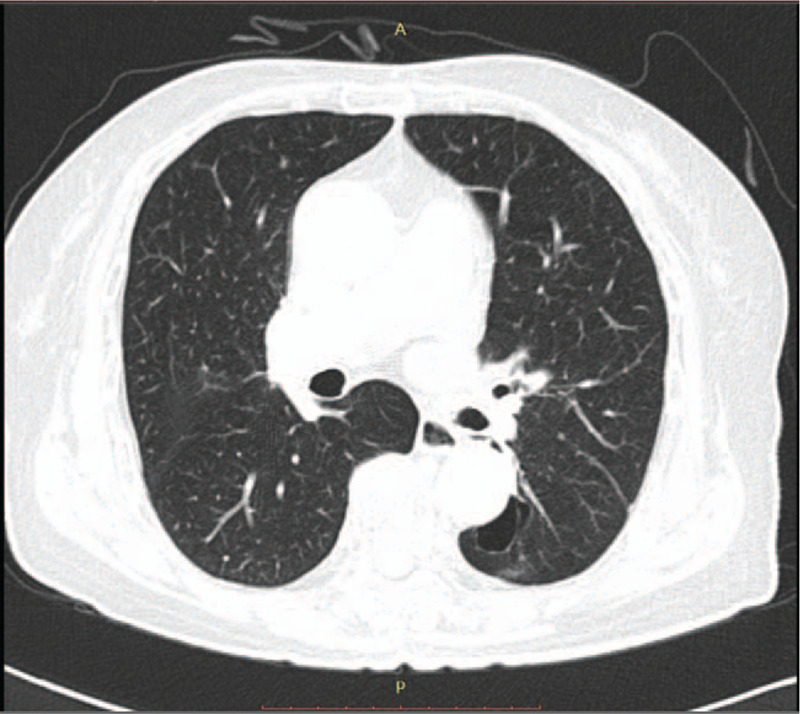
A recent chest computed tomography scan on June 8, 2020, indicated that the partial response was sustained at 78 months on the combined gefitinib and herbal medicine treatment.

**Figure 7:**
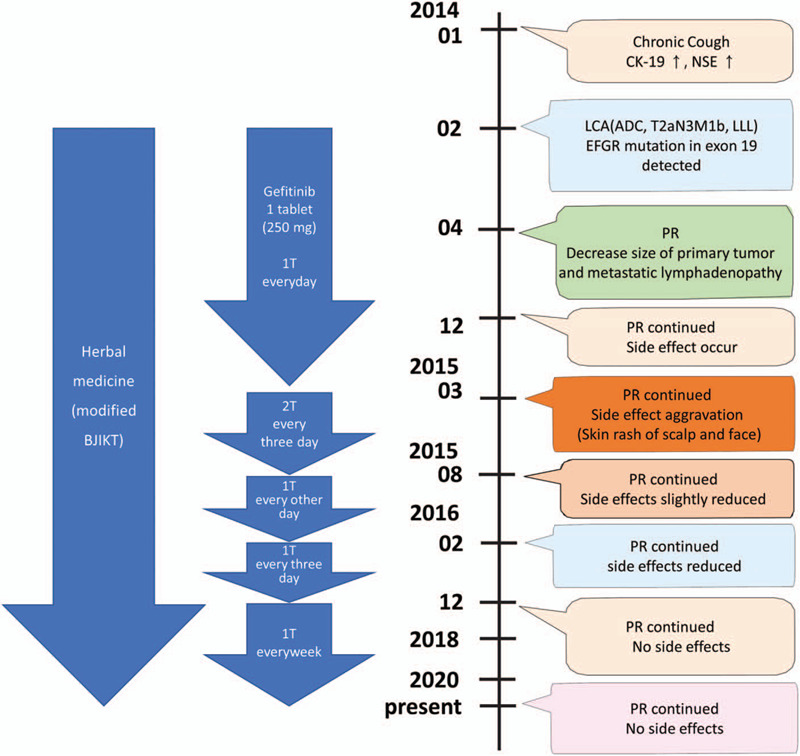
Timeline of interventions and outcomes. ADC = adenocarcinoma, BJIKT = Bojungikki-tang, CK-19 = cytokeratin 19, LCA = lung cancer, NSE = neuron-specific enolase, PR = partial response.

However, because the skin rash had worsened further to a grade 3 despite the previous dose reduction, the gefitinib dose was reduced to 1 tablet every other day. At that point, the skin rash started to diminish. The skin rash was reduced to grade 2 in February 2016, but still persisted; the gefitinib dose was then reduced to 1 tablet every 3 days. The PR continued in December 2016, the skin rash disappeared, and gefitinib was tapered to 1 tablet every week. Since then, the PR has been sustained (Fig. 6) without progression; the combination treatment of 1 tablet of gefitinib and the THM were maintained until June 2020 (Fig. 7).

## 3 Discussion

In this case study, we reported the case of an elderly patient with stage IV NSCLC who received continual treatment with a combination of gefitinib and modified BJIKT. She showed a sustained PR and PFS for over 78 months, although we gradually lowered the gefitinib dose due to a rash.

On the first visit, she was malnourished due to anorexia, with a body mass index (BMI) of 16.4 kg/m^2^. Her initial symptom was a cough; she was subsequently diagnosed with stage IV NSCLC. The prognostic factors for stage III–IV NSCLC Korean patients are the presence of symptoms, age, sex, BMI, and performance status.^[25]^ Therefore, a poor prognosis was expected in this patient because she had an initial symptom of cough, was >65 years old, and had a BMI <18.5 kg/m^2^.

The EGFR genotyping analysis revealed an exon 19 deletion in the EGFR mutation. Gefitinib was chosen for treatment because EGFR-TKIs are the standard first-line treatment for advanced NSCLC with an EGFR mutation.^[5]^ In general, gefitinib results in better PFS and OS in advanced NSCLC patients with an exon 19 deletion, and it is more effective in non-smoking Asian females with adenocarcinoma.^[6]^ Thus, gefitinib was expected to be effective.

At the same time, the THM was used simultaneously with gefitinib. Many studies on the combination of THM and conventional chemotherapy have shown synergistic effects in lung cancer.^[11,13]^ In particular, several clinical reports have indicated that the combination of adjuvant THM and EFGR-TKI, such as gefitinib, in patients with EGFR mutation-positive advanced NSCLC has shown good results, increasing OS and PFS and reducing side effects.^[12–14]^

In an experimental study, BJIKT had anti-cancer effects in lung cancer by increasing the rate of apoptosis^[21,26]^ and increasing the therapeutic effect of conventional chemotherapy.^[23]^ A clinical study reported that BJIKT was effective as an adjuvant treatment, enhancing conventional treatment in patients with NSCLC.^[24,27]^ Concern has been raised that the combination of THM and gefitinib may affect the pharmacokinetics of gefitinib, but BJIKT has been experimentally demonstrated to have no pharmacokinetic interaction with gefitinib.^[15]^

This patient exhibited several good responses to the combination therapy. First, the PR has been sustained for more than 78 months. PR was defined as a reduction in tumor size by 30%.^[28]^ In this case, the tumor size decreased by 61.5% from 3.9 cm to 1.5 cm, which was categorized as PR. Second, the brain metastatic lesions disappeared. Gefitinib has been reported to be effective for treating brain metastases in NSCLC, as it can pass the brain–blood barrier.^[29]^ According to this result, the combined treatment may have had a synergistic effect, and it apparently did not affect the pharmacokinetics of gefitinib. Third, the tumors were transformed into thin-wall cavities on CT after a certain period of time, a good prognostic factor. As a novel CT response, the presence of cavitation and attenuation changes within a target lesion can be used for response evaluation in patients with NSCLC who have undergone EGFR-TKI therapy, as cavitation within a tumor is caused by hampered angiogenesis and subsequent tumor necrosis. These morphological changes in the lesion are significantly associated with prolonged patient OS.^[30,31]^

However, a skin rash occurred after 1-year of gefitinib treatment and was getting worse, which deteriorated the patient's quality of life; thus, the gefitinib dose was reduced. Skin rash is a main side effect of EGFR-TKI therapy, and approximately two-thirds of NSCLC patients treated with EGFR-TKI experience dermatological toxicity, including skin rash. Skin rash is usually not lethal, but if it is severe it can necessitate a reduction in the dose of an anticancer agent.^[32]^ Therefore, treatment strategies with less toxicity are needed for elderly patients. A skin rash itself is a good prognostic factor,^[33]^ and retrospective studies have indicated that the PFS and OS of patients on low doses of gefitinib as a result of a dose reduction due to side effects are not inferior to those of patients who remain on the standard dose.^[34,35]^ However, the effect of reducing the gefitinib dose on clinical outcomes has not been established. In addition, low-dose gefitinib can induce early resistance.^[36]^ Therefore, low-dose gefitinib is not recommended unless side effects are severe.^[35]^ This patient has maintained PFS, although the dose of gefitinib was reduced 6 years ago.

In the Asian population with advanced NSCLC, gefitinib as first-line chemotherapy is associated with a median PFS of 5.7 to 10.4 months.^[6]^ Most patients with a good initial response to gefitinib develop acquired resistance within 6–12 months, resulting in tumor progression, which limits PFS.^[8]^ The clinical definition of acquired resistance to EGFR-TKIs is a systemic progression of the disease (according to the Response Evaluation Criteria in Solid Tumors or WHO criteria) while on continuous treatment with gefitinib within the last 30 days.^[7]^ However, in the present patient, PFS had been maintained for more than 78 months. Therefore, it is possible that the modified BJIKT delayed the emergence of acquired resistance to EGFR-TKI. However, re-biopsy for T790 M mutation, the main mechanism of acquired resistance, could not be performed for ethical reasons, as the patient did not have a progressive disease. Thus, a careful experimental study and clinical trials are required to confirm the delay of resistance.

This case report described the effect of a combined treatment of gefitinib and THM, a modified BJIKT. Despite the reduction in the gefitinib dose due to side effects, the combined treatment has maintained PFS for more than 78 months. Thus, we conclude that THM enhanced the anti-cancer effect of gefitinib in a patient with advanced NSCLC harboring the EFGR mutation; furthermore, it might have delayed acquired resistance, which is the main mechanism limiting the efficacy of gefitinib. However, further cases and prospective clinical studies are required to verify the efficacy.

### 3.1 Patient perspective

“The most difficult thing as a lung cancer patient is that my quality of life was reduced due to the side effects of chemotherapy. Although the severe rash made me reduce the dose of the anti-cancer drug, I was able to accept it peacefully because I believed that THM complemented the anti-cancer effect. After reducing the dose, the side effects stopped, and the anti-cancer effect continued. I feel comfortable and at ease in my daily life now.”

## Acknowledgments

This research was supported by a grant from the Korea Health Technology R&D Project through the Korea Health Industry Development Institute (KHIDI), funded by the Ministry of Health and Welfare, Republic of Korea (grant no. HI18C1944)

## Author contributions

**Conceptualization:** Jun-Hwan Lee.

**Data curation:** Cheong-Woon Choi.

**Funding acquisition:** Beom-Joon Lee, Kwan-Il Kim.

**Resources:** Cheong-Woon Choi.

**Supervision:** Jun-Hwan Lee.

**Validation:** Jun-Hwan Lee.

**Visualization:** Beom-Joon Lee, Kwan-Il Kim.

**Writing – original draft:** Beom-Joon Lee, Kwan-Il Kim.

**Writing – review & editing:** Beom-Joon Lee, Jong Yeol Kim.
